# Isolation and Genome Analysis of *Pectobacterium colocasium* sp. nov. and *Pectobacterium aroidearum*, Two New Pathogens of Taro

**DOI:** 10.3389/fpls.2022.852750

**Published:** 2022-04-26

**Authors:** Jianuan Zhou, Ming Hu, Anqun Hu, Chuhao Li, Xinyue Ren, Min Tao, Yang Xue, Shanshan Chen, Chongzhi Tang, Yiwu Xu, Lianhui Zhang, Xiaofan Zhou

**Affiliations:** ^1^Guangdong Province Key Laboratory of Microbial Signals and Disease Control, Integrative Microbiology Research Centre, South China Agricultural University, Guangzhou, China; ^2^Guangdong Tianhe Agricultural Means of Production Co., Ltd., Guangzhou, China; ^3^Qingyuan Agricultural Science and Technology Service Co., Ltd., Qingyuan, China

**Keywords:** taro soft rot, *P. colocasium* sp. nov., *P. aroidearum*, co-infection, genome sequencing

## Abstract

Bacterial soft rot is one of the most destructive diseases of taro (*Colocasia esculenta*) worldwide. In recent years, frequent outbreaks of soft rot disease have seriously affected taro production and became a major constraint to the development of taro planting in China. However, little is known about the causal agents of this disease, and the only reported pathogens are two *Dickeya* species and *P. carotovorum*. In this study, we report taro soft rot caused by two novel *Pectobacterium* strains, LJ1 and LJ2, isolated from taro corms in Ruyuan County, Shaoguan City, Guangdong Province, China. We showed that LJ1 and LJ2 fulfill Koch’s postulates for taro soft rot. The two pathogens can infect taro both individually and simultaneously, and neither synergistic nor antagonistic interaction was observed between the two pathogens. Genome sequencing of the two strains indicated that LJ1 represents a novel species of the genus *Pectobacterium*, for which the name “*Pectobacterium colocasium* sp. nov.” is proposed, while LJ2 belongs to *Pectobacterium aroidearum*. Pan-genome analysis revealed multiple pathogenicity-related differences between LJ1, LJ2, and other *Pectobacterium* species, including unique virulence factors, variation in the copy number and organization of Type III, IV, and VI secretion systems, and differential production of plant cell wall degrading enzymes. This study identifies two new soft rot Pectobacteriaceae (SRP) pathogens causing taro soft rot in China, reports a new case of co-infection of plant pathogens, and provides valuable resources for further investigation of the pathogenic mechanisms of SRP.

## Introduction

Soft rot Pectobacteriaceae (SRP) consists of phytopathogens in the genera of *Pectobacterium* and *Dickeya*, and its members are distributed all over the world, especially on angiosperm plants in geographically diverse regions (Ma et al., 2007; Toth et al., 2021b). *Pectobacterium* and *Dickeya* are characterized as opportunistic pathogens that switch from an asymptomatic latent phase into a virulent phase under suitable environmental conditions (Pérombelon, 2002; Lebeau et al., 2008). Since SRP relies mainly on the secretion of cell wall degrading enzymes (CWDEs) provoking maceration symptoms, these bacteria have long been considered as “brute-force” necrotrophic pathogens (Toth and Birch, 2005).

The main virulence determinants of SRP include the type I–VI secretion systems (T1SS–T6SS). Known types of T1SS include an ABC-type PrtDEF T1SS responsible for the secretion of proteases (Charkowski et al., 2012), a LssBEF T1SS functioning as bacteriocin exporter, a LapBCDE T1SS allowing the secretion of proteinaceous multi-repeat adhesin attaching to root surfaces (Bell et al., 2004; Pérez-Mendoza et al., 2011), and a HasDEF T1SS transporting siderophore (Bell et al., 2004; Gorshkov et al., 2018). The T2SS Out secretion system is responsible for the translocation of most pectinases and cellulases (Charkowski et al., 2012), necrosis-inducing protein NipE (Mattinen et al., 2004), and AvrL-AvrM proteins (Kazemi-Pour et al., 2004). In addition, the twin-arginine translocation (Tat) protein system exports the pectin lyase PnlH, which is important for the virulence and fitness of *Dickeya* pathogens (Coulthurst and Palmer, 2008; Rodríguez-Sanz et al., 2010; Zhang et al., 2018). T3SS is present in most *Pectobacterium* spp. and play a role in disease development and plant immunity (Holeva et al., 2004; Kim et al., 2011). T4SS is the only system able to transport nucleic acids in addition to proteins into host cells (Christie and Cascales, 2005). Inactivation of T4SS resulted in a weak defect in virulence of *P. atrosepticum* (Bell et al., 2004). T5SS and T6SS are both contact-dependent competition systems, secreting a large number of proteins to mediate stress responses for bacterial environmental survival and host adaptation (Mattinen et al., 2007; Liu et al., 2008; Bellieny-Rabelo et al., 2019; Yu et al., 2021).

Other virulence factors contributing to *Pectobacterium* pathogenicity include flagella-based motility, cell membrane structures such as enterobacterial common antigen (ECA) and lipopolysaccharide (LPS), necrosis-inducing protein (Nip), coronafacic acid (CFA), plant ferredoxin-like protein (FerE), citrate uptake, 3-hydroxy-2-butanone pathway, and carotovoricin (CTV) (Bell et al., 2004; Mattinen et al., 2004; Toth and Birch, 2005; Yamada et al., 2006; Urbany and Neuhaus, 2008; Evans et al., 2010; Marquez-Villavicencio et al., 2011b; Charkowski et al., 2012; Bellieny-Rabelo et al., 2019).

The *Pectobacterium* genus now contains 20 species (Moussa et al., 2021; Toth et al., 2021a), including *P. actinidiae* (Koh et al., 2012; Portier et al., 2019), *P. aquaticum* (Pédron et al., 2019), *P. aroidearum* (Yishay et al., 2008; Nabhan et al., 2013), *P. atrosepticum* (Gardan et al., 2003), *P. betavasculorum* (Thomson et al., 1981; Gardan et al., 2003), *P. brasiliense* (Nabhan et al., 2012; Portier et al., 2019), *P. cacticida* (Alcorn et al., 1991), *P. carotovorum* (Skerman et al., 1980; Portier et al., 2019), *P. fontis* (Oulghazi et al., 2019), *P. odoriferum* (Gallois et al., 1992; Gardan et al., 2003; Portier et al., 2019), *P. parmentieri* (Khayi et al., 2016), *P. parvum* (Pasanen et al., 2020), *P. peruviense* (Waleron et al., 2018), *P. polaris* (Dees et al., 2017), *P. polonicum* (Waleron et al., 2019), *P. punjabense* (Sarfraz et al., 2018), *P. quasiaquaticum* (Moussa et al., 2021), *P. versatile* (Portier et al., 2019), *P. wasabiae* (Goto and Matsumoto, 1987), and *P. zantedeschiae* (Waleron et al., 2019).

*Pectobacterium* pathogens have been reported to have overlapping host ranges and, in many cases, are distributed in the same geographical areas (van der Wolf et al., 2021). However, there are only a few reports on the isolation of multiple pathogens from the same soft rot samples (Caruso et al., 2016; Elhalag et al., 2020). For example, according to the analysis of the prevalence of *Dickeya* spp. And *Pectobacterium* spp. On seed potato plantations in Poland in 2013 and 2014, 35.5% (2013) and 15.1% (2014) of all tested samples were co-infected by more than one pectolytic pathogens of *P. atrosepticum*, *P. carotovorum*, *P. parmentieri*, and *Dickeya* spp. (Motyka-Pomagruk et al., 2021). The interactions between pathogens in such mixed infection events are even less studied (Marquez-Villavicencio et al., 2011a; Ge et al., 2021).

Pinang taro (*Colocasia esculenta* L. var. *cormosus* Chang), which belongs to the Araceae family, is a large taro with a plant height of over 2 m and bulb’s weight up to 4∼6 kg. It has high nutritive and economic value and is mainly planted in Guangdong, Fujian, Guangxi, Hunan, and Hainan provinces in China (Guo and He, 2015; Dong et al., 2019; Que, 2020). Guangdong Province alone has a planting area of 13,000 hectares, with an annual yield of 292.5 kilotons, generating an annual output value of more than 0.47 billion RMB.^1^ Soft rot is one of the most serious diseases of taro. In recent years, the occurrence of soft rot in taro planting areas has been increasing, and some fields even failed to harvest, posing a severe threat to the production of taro in China (Dai and Yu, 2011). However, our understanding of the disease remains limited. Farmers and plant protection workers often confuse it with taro leaf blight caused by *Phytophthora* pathogens and carry out an ineffective fungicide treatment.

It is critical to know the pathogens that cause the disease first for successful disease control. It has long been recognized that taro soft rot can be caused by both bacterial (*D. chrysanthemi*) and oomycete (*Pythium* spp.) pathogens. Recently, two additional members of the *Dickeya* genus, namely *D. fangzhongdai* and *D. zeae*, were reported to be pathogenic to taro (Boluk et al., 2021; Huang et al., 2021). Besides, *P. carotovorum* has also been stated as one of the taro pathogens with no original experimental isolation evidence (Wu and Dai, 2015; Dong et al., 2021).

Ruyuan County is located at 24°28′–25°09′ north latitude and 112°52′–113°28′ east longitude, in the south part of Guangdong Province and the west of Shaoguan City, with typical subtropical monsoon climate. It borders Lechang City in the north, where taro soft rot outbreaks have been reported recently (Huang et al., 2021). The planting area of taro in Ruyuan County is about 200 hectares. Taro is intercropped with water chestnut and rice. Soft rot is the top disease, followed by taro leaf blight in Ruyuan. Almost all taro plants in the fields suffer the disease with different incidence rates from April to October, with summer (June to September) being the most severe period. In worst cases, less than one-third of taro corms can be harvested.

In this study, we aimed to isolate the pathogenic bacteria responsible for soft rot disease of taro planting in Ruyuan County, China. Two new taro soft rot pathogens, LJ1 representing a novel species of *Pectobacterium* for which we propose to name as “*P. colocasium* sp. nov.,” and LJ2, which belongs to *P. aroidearum*, were identified. We carried out pathogenicity tests and co-infection assays to characterize the pathogens and their potential interactions. Furthermore, we sequenced their genomes and performed genomic comparisons among LJ1, LJ2, and other pectobacteria to better understand the features of these taro pathogens. The findings of this study provide an important basis for further investigations on the distribution of the pathogens and the epidemiological significance of the identified strains.

## Materials and Methods

### Disease Samples Collection and Microbial Isolation

On 13th October 2019, four corms of taro plants showing wilting leaves were collected from Laojun Village, Ruyuan County, Shaoguan City, Guangdong Province, China. Microbial separation from the symptomatic taro was carried out using the method previously described (Hu et al., 2021b). Firstly, the corms were washed with tap water and then surface-disinfected with 70% ethanol solution. Secondly, the corms were cut open using a sterile knife in the biosafety cabinet. Then, rotten tissue was picked out from the center part with a sterile inoculation loop and streaked onto a Luria-Bertani (LB, containing 10 g/L typtone, 5 g/L yeast extract and 10 g/L NaCl) agar (1.5% w/v) plate for incubation at 28°C for 24 h. Finally, single colonies were streaked onto fresh medium plates for purification and grown in LB medium with shaking at 200 rpm overnight for preservation.

### 16S rDNA Gene Sequencing of Single Colonies

Bacteria were grown in LB medium until OD_600_ of 1.0, and genomic DNAs were extracted using the EasyPure Bacteria Genomic DNA Kit (TransGen Biotech, Beijing, China). The 16S rDNA gene sequences were amplified using the primers 27f and 1492r (Coenye et al., 1999). The products were examined using 1% agarose gel electrophoresis and sent to Sangon Biotech Company in Shanghai, China, for sequencing. SeqMan V.5.00 was used to assemble sequences generated from forward and reverse primers.

### Pathogenicity Tests of the Isolated Strains

To test whether LJ1 and LJ2 could cause soft rot in the field, we carried out potted experiments with wound and non-wound inoculation. Bacterial strains were first cultured to the logarithmic phase till OD_600_ of 1.2 in LB medium. For wound inoculation, 100 μL of LJ1, LJ2, and LJ1 + LJ2 mixture were, respectively, injected into the taro seedling tuberous roots. LB medium was used as a negative control. For non-wound inoculation, 10 mL of LJ1, LJ2, and LJ1 + LJ2 mixture were, respectively, centrifuged, re-suspended with 50 mL of PBS (Phosphate Buffer Solution), and then poured into the potted plants. PBS buffer was used as the negative control. All the pots were kept in a greenhouse (28 ± 2°C, 75 ± 15% relative humidity, and 12 h alternating light and dark cycles) and watered once a day with sterile water until disease symptoms were observed. The experiment was repeated three times in triplicate. To fulfill Koch’s postulates, the bacteria were re-isolated from the diseased slices compared with the corresponding sequences of the inoculated ones for their 16S rDNA gene sequences.

To test the pathogenicity of LJ1 and LJ2 on different plants, bacterial strains were grown in LB medium till OD_600_ of 1.2, and different plant organs were selected using different inoculation methods. *P. carotovorum* BC9 was used as a control. For potato (*Solanum tuberosm*), Chinese cabbage (*Brassica pekinensis*), celtuce *(Lactuca sativa* var. *angustata*), and onion (*Allium cepa*), tubers/stems were washed with tap water and dried with a paper towel. Potato and celtuce were subsequently surface-sterilized with 70% ethanol and then sliced evenly about 5 mm in thickness. Plant tissue was placed in a tray with moistened filter paper, and 2 μL of bacterial culture was applied to the center of the tissue pierced with pipette tips. For *Alocasia macrorrhiza*, 100 μL of bacterial culture was injected into the basal stems of the seedlings, and 5 μL onto the leaves. All trays were kept in a growth chamber (Shanghai YiHeng Scientific Instruments Co., Ltd., Shanghai, China) with controlled conditions as 28 ± 2°C, and 75 ± 15% relative humidity till symptoms appeared. Same volume of LB medium was inoculated as the negative control. Each assay was repeated three times with triplicate. The areas of lesions were measured using Image J 1.52s software (Schneider et al., 2012).

### Phenotypic Characteristics Analysis of Strains LJ1 and LJ2

Biochemical characteristics of strains LJ1 and LJ2 were determined using Biolog GEN III microplates (bioMérieux) following the manufacturer’s protocol. For chemotaxonomic analysis of fatty acids, biomasses of exponentially growing cells of LJ1 and LJ2 cultured in MB medium (TOPBIO, Shandong Tuopu Bioi-engineering Co., Ltd., Zhaoyuan, China) at 25°C for 3 days were harvested and lyophilized. Cellular fatty acids were saponified, methylated, and extracted according to the protocol of the Sherlock Microbial Identification System (MIDI) and identified by gas chromatography (model 7890B; Hewlett Packard, Santa Clara, United States) using the Microbial Identification software package with the MIDI Sherlock system and the Sherlock Aerobic Bacterial Database (RTSBA6 6.21).

### Test on the Relationship Between Isolates LJ1 and LJ2

To determine whether there is an antagonistic interaction between LJ1 and LJ2, we performed an antibacterial assay using the method previously described (Hu et al., 2018). In brief, single colonies of isolates LJ1, LJ2, and *Escherichia coli* DH5α on LB agar plates were transferred into LB medium for growth with shaking at 200 r/m at 28°C (or 37°C for *E. coli* DH5α) till OD_600_ of 1.2. To test the antagonism of LJ1 against LJ2, 1 mL of LJ2 bacterial culture was added into 20 mL of 1% melted agarose (cool to 50∼60°C), mixed thoroughly, and then laid onto a LB agar plate (10 cm × 10 cm). After solidifying, wells of 5 mm in diameter were punched and applied with 20 μL of LJ1 bacterial culture. Antagonistic assay of LJ2 against LJ1 was performed in the same way. LB medium was used as the negative control, and *P. carotovorum* BC9 reserved in our lab, which produces antibacterial substances, was used as the positive control. The plates were incubated at 28°C for 24 h. The experiment was repeated three times in triplicate.

To determine whether strains LJ1 and LJ2 promote or inhibit each other in planta, we measured the bacterial cell numbers by counting their corresponding colony-forming units (CFU) according to the method previously described (Hu et al., 2021a; Xue et al., 2021). Firstly, we visualized these two strains by, respectively, importing plasmids pBBP_*gdh*_ (pmCherry) (Liao et al., 2018) and pLAFR3-GFP (pGFP) (Shi et al., 2019) into strains LJ1 and LJ2 by triparental mating with the help of *E. coli* HB101(RK2013). Secondly, 10 μL of bacterial cultures (grown in LB medium till OD_600_ of 1.5) of LJ1(pBBP_*gdh*_), LJ2(pLAFR3-GFP), and LJ1(pBBP_*gdh*_) + LJ2(pLAFR3-GFP) mixture were, respectively, spotted onto taro slices. LB medium was spotted as the negative control. The slices were placed on Petri dishes, sealed and kept in a biological incubator under the conditions described above. After 24 and 48 h, the diseased tissue was cut off and weighed, and then ground into homogenate with 10 mL of PBS buffer. The homogenate was diluted in 10× series; 100 μL of each gradient dilution was spread onto fresh LB agar plates and incubated at 28°C overnight. Colonies on plates were counted (plates with less than 30 or more than 300 colonies were considered outliers and discarded). The experiment was repeated three times in triplicate.

### Genome Sequencing, Assembly, and Annotation

Genomic DNAs were extracted from LJ1 to LJ2 in LB medium culture using EasyPure^®^ Bacteria Genomic DNA Kit (Transgen, Beijing, China). The harvested DNAs were detected by the agarose gel electrophoresis and quantified by Qubit^®^ 2.0 Fluorometer (Thermo Scientific, Waltham, MA, United States). The complete genomes were sequenced by Grandomics Biosciences (Beijing, China) using the Nanopore PromethION platform and Illumina MGISEQ T7 platform. The genome assemblies were generated using Flye v2.7 with long-read data alone, which were then polished with both long- and short-read data by medaka v1.2.5 and pilon v1.2.3 (Wick et al., 2017), respectively. The sequencing data and genome assemblies have been deposited in the NCBI database under the accessions CP084032.1 (PRJNA765110) and CP084023.1 (PRJNA765112), respectively.

The circular visualization of LJ1 and LJ2 genome characteristics was created by Circos (Krzywinski and Schein, 2009). PGAP (2020-09-24.build4894) (Tatusova et al., 2016) was used for genome annotation. Virulence factors were predicted by PathoFact v1.0 with default settings (de Nies et al., 2021), and only the predictions with high confidence levels (i.e., “1: Secreted Virulence factor” and “2: Non-secreted Virulence factor”) were considered.

### Phylogenetic Analysis of LJ1 and LJ2 and Genomic Comparison of Representative Genomes of *Pectobacterium* Species

Pairwise average nucleotide identity (ANI) values between LJ1, LJ2, and all 327 *Pectobacterium* genomes available in the NCBI RefSeq database (Supplementary Table 1) were calculated using fastANI v1.3. Digital DNA-DNA hybridization (dDDH) values between LJ1, LJ2, and *Petobacterium* genomes that share the highest ANI values with LJ1 or LJ2 were then determined by using the GGDC server.^2^ Orthologous gene groups were constructed using OrthoFinder v2.5.4 from annotated proteins in the genomes of LJ1, LJ2, and representative genomes of the 20 *Pectobacterium* species. The OrthoFinder analysis was conducted with default settings except that the sensitivity of diamond search was set to “ultrasensitive.” The orthologous groups defined in the “Orthogroups.tsv” result file were used in downstream analyses such as the identification of unique genes or orthologs of previously reported SRP genes encoding secretion systems and CWDEs (Bell et al., 2004; Coulthurst and Palmer, 2008; Rodríguez-Sanz et al., 2010; Pérez-Mendoza et al., 2011; Zhou et al., 2015; Gorshkov et al., 2018; Zhang et al., 2018). The genome annotations and orthologous groups generated in this study have been deposited in the FigShare repository and available at https://figshare.com/projects/Isolation_and_genome_analysis_of_Pectobacterium_colocasium_sp_nov_and_P_aroidearum_two_new_pathogens_of_taro/134858. A phylogenetic analysis of LJ1, LJ2, and the representative *Pectobacterium* genomes was inferred based on a set of 1,621 single-copy orthologous genes present in most analyzed genomes. Single-gene alignments were generated using MAFFT v7.490 (with the “E-INS-i” iterative refinement method), trimmed for gappy columns using trimAl v1.4 (with the “gappyout” option), and then concatenated into one supermatrix. The phylogenetic inference was performed using IQ-TREE v2.1.2 with an automatic selection of the best-fitting evolutionary model for each gene, and the reliabilities of internal branches were estimated by ultrafast bootstrap analysis with 1,000 replicates.

### Measurement of Cell Wall Degrading Enzymatic Activities

The activities of cell wall degrading enzymes (CWDEs) were measured using the assay medium recipe described previously (Zhou et al., 2016). Briefly, 30 mL of each assay medium containing enzyme reaction substrate was poured into a 13 × 13 cm square plate. After solidification, wells (5 mm in diameter) were punched, and 20 μL of bacterial cells (OD_600_ of 1.5) were added into the wells. All the assay plates were incubated at 28°C. After 11 h, the pectate lyase (Pel) assay plate was covered with 4 M HCl; after 14 h, the cellulase (Cel) assay plate was stained with 0.1% (w/v) Congo Red for 15 min and discolored with 1 M NaCl twice. Protease (Prt) activity was indicated by the transparent halos surrounding the wells after 24 h without any treatment. The experiment was repeated three times in duplicate. GraphPad Prism 8.4.1 was used to perform the unpaired two-tailed *t*-test.

## Results

### Disease Symptoms of Bacterial Soft Rot of Taro in Ruyuan County

**FIGURE 1:**
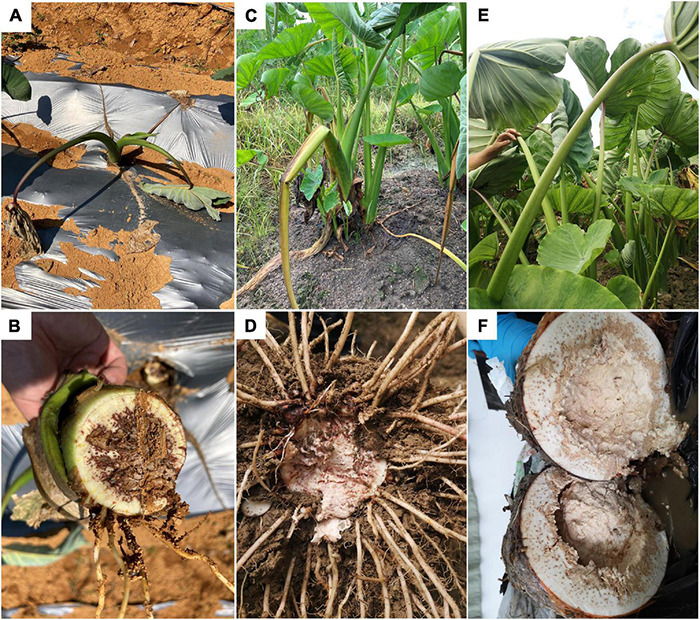
Symptoms of bacterial soft rot of Pinang taro in the field. **(A)** Diseased plants in early (0–3 months old) seedling stages; **(B)** Basal stem of a diseased plant in the early seedling stage; **(C)** Diseased plants in middle (4–6 months old) seedling stage; **(D)** Diseased bulb in middle seedling stage; **(E)** Diseased plants in late (7–8 months old) seedling stage; **(F)** Diseased bulb in late seedling stage.

This disease happens in all growing stages. In the early seedling stage (1–5 leaves, 0–3 months old), the plant shows green wilting with drooped down leaves (Figure 1A); inside the basal pseudostem, brown rot with smelly bacterial pus can be seen in vascular bundles, the edge of the lesion is dry, and the middle is wet and rotten (Figure 1B). In the middle stage (6–8 leaves, 4–6 months old), leaves of the infected plant curl up, some lodge (Figure 1C), and bean curd-like rotten tissue can be observed in the corm (Figure 1D). In the harvest stage (corm growing, 7–8 months old), the infected plant wilts, leaves remain green but become frizzy (Figure 1E). The corm has a strong odor, and the internal tissue decays completely after splitting (Figure 1F). The petiole and leaf blade remain green throughout the disease course, indicating that pathogens preferentially colonize underground corms.

### Pathogen Isolation and Inoculation

On 13th October 2019, four corms of taro plants showing wilting leaves were collected from Laojun Village, Ruyuan County, Shaoguan City, Guangdong Province, China. Rotten tissues of the soft rot corms were used for pathogen separation. As a result, 18 isolates in total (names as LJ1–LJ18) were isolated from the tissue, which were putatively classified as *Pectobacterium aroidearum* (eight isolates), *Bacillus* sp. (four isolates), *Klebsiella* sp. (three isolates), *P. carotovorum* (two isolates), and *Weissella cibaria* (one isolate) based on 16S rDNA sequence analysis (Supplementary Table 1).

**FIGURE 2:**
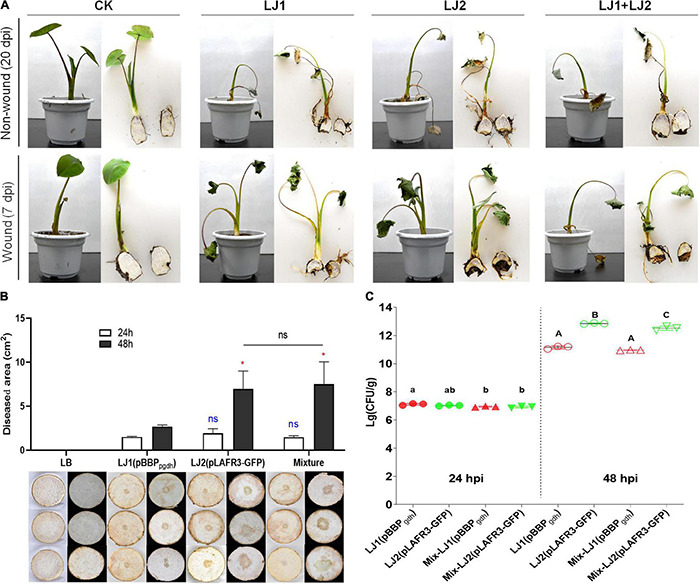
Pathogenicity tests of strains LJ1 and LJ2 on taro. **(A)** Non-wound (irrigation) and wound (injection) inoculation of strains LJ1 and LJ2 on taro seedlings. For non-wound inoculation, 10 mL of LJ1, LJ2, and LJ1 + LJ2 mixture (OD_600_ of 1.2) were, respectively, centrifuged, re-suspended with 50 mL of PBS, and poured into the potted plants. PBS buffer was used as the negative control. The plants were kept in a greenhouse (28 ± 2°C, 75% ± 15% relative humidity, and 12 h alternating light and dark cycles) and watered once a day with sterile water until disease symptoms were observed. For wound inoculation, 100 μL of LJ1, LJ2, and LJ1 + LJ2 mixture (OD_600_ of 1.2) were, respectively, injected into the taro seedling tuberous roots. LB medium was used as a negative control. **(B)** Symptoms of taro soft rot after 24 and 48 h post-inoculation (hpi) with LB, strain LJ1, strain LJ2, and LJ1 + LJ2 mixture, respectively. Strains LJ1 and LJ2 were first, respectively, visualized by introducing fluorescence plasmids pBBP_*gdh*_ (pmCherry) (Liao et al., 2018) and pLAFR3-GFP (pGFP) (Shi et al., 2019), and then 10 μL of bacterial cultures (grown in LB medium till OD_600_ of 1.5) of LJ1(pBBP_*gdh*_), LJ2(pLAFR3-GFP), and LJ1(pBBP_*gdh*_) + LJ2(pLAFR3-GFP) mixture were, respectively, spotted onto taro slices. LB medium was spotted as the negative control. The slices were placed on Petri dishes, sealed and kept in a biological incubator as conditions described above and observed. Photos were taken after 24 and 48 h post-inoculation. The experiment was performed in triplicate. GraphPad Prism 8.4.1 was used to perform unpaired two-tailed *t*-test, and the data of LJ2 and LJ1 + LJ2 were compared with those of strain LJ1 at corresponding post-inoculation time (blue for 24 h, red for 48 h). Data of LJ1 + LJ2 were also compared with those of LJ2 (black). The data present the means of three replicates and error bars represent the standard deviation. “ns” indicates not significant, *P < 0.05. **(C)** The colony-forming units (CFU) of LJ1 and LJ2 in taro tissue after 24 and 48 (hpi). After 24 and 48 h of inoculation on taro slices, the diseased tissue was cut off and weighed, and then ground into homogenate with 10 mL of PBS buffer. The homogenate was diluted in series, and 100 μL of each gradient dilution was spread onto fresh LB agar plates, incubated at 28°C overnight. Colonies between 30 and 300 in the plates were counted. The experiment was repeated three times in triplicate. Statistical analysis was performed on each group of data and significantly different values (ANOVA *P* < 0.05) are indicated by different letters.

Given that *Pectobacterium* spp. can cause soft rot disease in many kinds of crops and vegetables, we selected two of the *Pectobacterium* isolates for pathogenicity tests, namely LJ1 and LJ2, which were closest to *P. aroidearum* SCRI 109 and *P. aroidearum* L6, respectively. Inoculums of pure LJ1, pure LJ2, and LJ1 + LJ2 mixture were each applied to taro seedlings with both non-wound (irrigation) and wound (injection) inoculation methods. At 20 days post-inoculation (dpi), wilting symptoms were observed in taro plants irrigated with LJ1, LJ2, or their mixture. Similar symptoms were visible at 7 dpi in injected plants (Figure 2A). Brown rotten tissue could be observed after longitudinal incision of the corms of inoculated plants, whereas all control taro plants remained symptom-free (Figure 2A). The pathogens were further re-isolated from the diseased tissue and confirmed as the same original strains by 16S rDNA sequencing, thus fulfilling the Koch’s postulates.

To explore the host range of LJ1 and LJ2, we also performed pathogenicity tests on potato, Chinese cabbage, celtuce, onion, and *Alocasia macrorrhizos*. Results showed that both strains can also infect these plants except onion; LJ2 was more virulent than LJ1 on Chinese cabbage and celtuce, while both caused only slight disease symptoms on the leaves of *A. macrorrhizos* (Supplementary Figure 1).

### Interaction Between Isolates LJ1 and LJ2

Since LJ1 and LJ2 were isolated from the same diseased corm, we further examined whether there is any interaction between them. Firstly, antagonistic activity between the two isolates was measured, and results showed no growth inhibitory halo around the wells applied with LJ1 (or LJ2) culture on the bacterial lawn plate of LJ2 (or LJ1) (Supplementary Figure 2). Furthermore, we inoculated both taro corm slices and plants with mCherry-labeled LJ1, GFP-labeled LJ2, or the mixture of both to assess the interaction of the two strains within host by evaluating disease severity and cell propagation. Twenty-four hours post-inoculation, both individual inoculations of either strain and the co-inoculation of LJ1 + LJ2 resulted in macerated taro corm slices with similar lesion areas (Figure 2B). Forty-eight hours post-inoculation, LJ2 alone and the LJ1 + LJ2 mixture resulted in comparable lesions significantly larger than the lesion caused by LJ1 (Figure 2B). In concordance with this data, LJ1 and LJ2 exhibited comparable numbers of CFU in decayed tissues in both pure inoculation and co-inoculation at 24 dpi, whereas LJ2 became considerably more abundant than LJ1 in both conditions at 48 dpi (Figure 2C). Notably, LJ1 was only slightly more abundant (1.47-fold) in pure inoculation than in co-inoculation at 24-hpi, and exhibited comparable CFU numbers in both conditions at 48-hpi (Figure 2C). A similar trend was observed for LJ2, which had similar CFU numbers at 24-hpi, and was 2.07-fold more abundant in pure inoculation than in co-inoculation at 48-hpi (Figure 2C). Overall, the results of co-infection assays suggest that LJ1 and LJ2 do not interact with each other.

### Genome Sequencing and Analysis of Strains LJ1 and LJ2

To better understand the molecular basis of their pathogenicity, the genomes of LJ1 and LJ2 were sequenced using both Nanopore PromethION platform and Illumina NovaSeq platform. The genomes of LJ1 and LJ2 were both assembled into single, circular chromosomes of 4,912,018 bp and 4,857,800 bp in size, and 51.65 and 51.87% in GC-content, respectively (Supplementary Figure 3 and Table 1). A total of 4,316 and 4,255 protein-coding genes were predicted in the genomes of LJ1 and LJ2, respectively; the two strains both have 108 RNA genes, all of which are conserved except one unique ncRNA gene in LJ1 and one unique tRNA gene in LJ2. Interestingly, LJ1 contains substantially more transposases (71:21) and pseudogenes (122:70) than LJ2 (Table 1).

**TABLE 1:** Genomic features of LJ1 and LJ2.

Features	LJ1	LJ2
Size (bp)	4,912,018	4,857,800
GC content (%)	51.65	51.87
Gene	4,424	4,363
CDS	4,316	4,255
RNA genes	108	108
rRNA	22	22
tRNA	77	78
ncRNA	9	8
Transposase	71	21
Pseudogene	122	70
CRISPR	2	0

To determine the taxonomic classifications of LJ1 and LJ2, we compared their genomes with the 327 *Pectobacterium* genomes available in the NCBI RefSeq database and a custom assembly of *P. cacticida* ICMP 11136 (see Section “Materials and Methods”), which altogether represent all the 20 known *Pectobacterium* species. We first calculated the average nucleotide identity (ANI) values between all genomes. The results showed that LJ1 is most closely related to *Pectobacterium* sp. CFBP8739 (GCF_013449375.1, isolated from irrigation river water in France) with an ANI value of 93.68% (Supplementary Table 2) and a dDDH value of 52.00%, both below the commonly accepted thresholds (ANI ≥ 95%, and dDDH ≥ 70%) for species delineation. For LJ2, the closest relative is *P. aroidearum* L6 (GCF_015689195.1) with an ANI value of 98.33% (Supplementary Table 2) and a dDDH value of 84.50%, indicating that LJ2 belongs to *P. aroidearum*.

**FIGURE 3:**
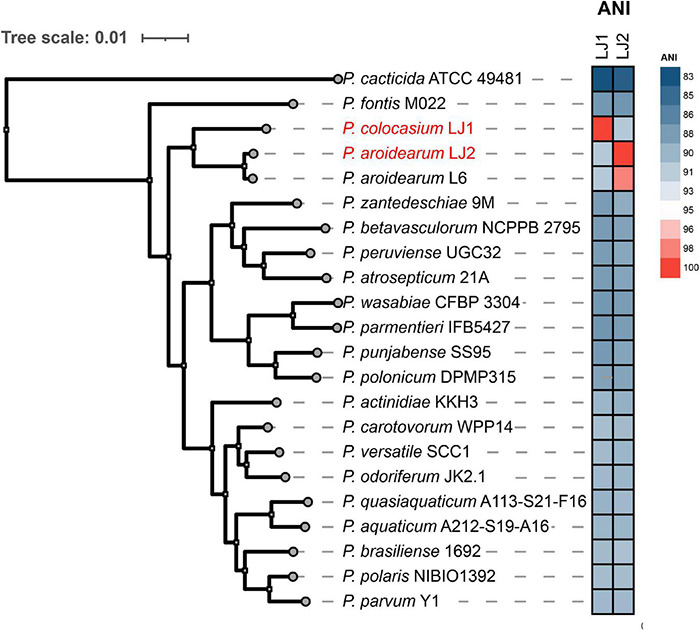
Phylogenetic analysis of *Pectobacterium colocasium* sp. nov. LJ1, *Pectobacterium aroidearum* LJ2, and representative strains of the 20 known *Pectobacterium* species. The phylogeny on the left is a maximum-likelihood tree inferred using IQ-TREE based on a concatenation-based analysis of 1,621 single-copy genes identified in the *Pectobacterium* pan-genome analysis. All internal branches in the tree received 100% bootstrap support. A heatmap of the ANI values between LJ1/LJ2 and the 20 representative *Pectobacterium* strains is shown in the middle.

We then performed a phylogenomic analysis to investigate further the relationships between LJ1, LJ2, and representative strains of the 20 known *Pectobacterium* species. The phylogeny showed that LJ2 and *P. aroidearum* L6 are sister to each other and very closely related, corroborating the assignment of LJ2 to *P. aroidearum*. These two *P. aroidearum* strains are then grouped with LJ1; however, they are separated by a longer evolutionary distance in the tree than that between LJ2 and J6, consistent with the relatively low ANI values between LJ1 and LJ2 (91.35%) or L6 (91.40%) (Supplementary Table 2 and Figure 3). All other *Pectobacterium* species are more distantly related to LJ1/LJ2.

Since our genomic analyses indicate that LJ1 might represent a novel species, we decided to perform biochemical and fatty acid analyses of LJ1 and LJ2. As shown in Table 2, the two strains exhibited several differences in their utilization of carbon sources, including that: (1) LJ1 could not use citric acid, while LJ2 could use; (2) LJ1 was weakly responsive to D-cellobiose, D-raffinose, D-melibiose, and Tween 40, while LJ2 was negative to them; and (3) LJ2 had a weak response to 3-methyl glucose, inosine, D-arabitol, and L-alanine, while LJ1 did not. Our chemotaxonomic analysis showed that the major (>5%) cellular fatty acids of strains LJ1 and LJ2 both included summed feature 3 (28.96 and 29.44%, respectively, comprising C_16:1_ω6*c* and/or C_16:1_ω7*c*), C_16:0_ (28.75 and 25.55%, respectively), summed feature 8 (18.06 and 19.47%, respectively, comprising C_18:1_ω6*c* and/or C_18:1_ω7*c*), summed feature 2 (10.39 and 9.29%, respectively, comprising C_14:0_ 3-OH, iso-C_16:1_ I and/or C_12:0_ aldehyde), and C_12:0_ (5.71 and 6.69%, respectively) (Table 2). In addition, C_17:0_ cyclo and C_15:1_ ω8*c* were detected in LJ2 but not LJ1 (Table 2). Both strains were most closely related to *P. carotovorum* in their chemotaxonomic profiles, but the similarity index of LJ1 (0.542) was considerably lower than that of LJ2 (0.673). The above phenotypic and genomic data all indicate that strain LJ1 should represent a novel species of the genus *Pectobacterium*, for which the name “*Pectobacterium colocasium* sp. nov.” is proposed after the species name of its natural host.

**TABLE 2:** Phenotypic characteristics of strains LJ1 and LJ2.

Characteristics	LJ1	LJ2
pH 5.0	W	W
pH 6.0	+	+
1% NaCl	+	+
4% NaCl	W	W
8% NaCl	−	−
Utilization of carbon sources (Biolog GEN III):		
Dextrin	−	−
D-Maltose	−	−
D-Trehalose	W	W
D-Cellobiose	W	−
Gentiobiose	+	+
Sucrose	+	+
D-Turanose	−	−
Stachyose	−	−
D-Raffinose	W	−
α-D-Lactose	+	+
D-Melibiose	W	−
β-Methyl-D-glucoside	+	+
D-Salicin	+	+
*N*-Acetyl-D-glucosamine	+	+
*N*-Acetyl-β-D-mannosamine	W	W
*N*-Acetyl-D-galactosamine	−	−
*N*-Acetyl neuraminic acid	−	−
α-D-Glucose	+	+
D-Mannose	+	+
D-Fructose	+	+
D-Galactose	+	+
3-Methyl glucose	−	W
D-Fucose	−	−
L-Rhamnose	W	+
Pectin	+	+
D-Galacturonic acid	+	+
L-Galactonic acid lactone	+	+
D-Gluconic acid	W	W
D-Glucuronic acid	W	W
Glucuronamide	W	W
Mucic acid	+	+
Quinic acid	−	−
D-Saccharic acid	+	+
p-Hydroxy-phenylacetic acid	−	−
Methyl pyruvate	+	W
L-Lactic acid	−	W
Citric acid	−	+
α-Keto-Glutaric acid	−	−
D-Malic acid	−	−
L-Malic acid	+	+
Bromo-succinic acid	+	+
Tween 40	W	−
γ-Amino-butryric acid	−	−
α-Hydroxy-butyric acid	−	−
β-Hydroxy-D,L-butyric acid	−	−
α-Keto-butyric acid	−	−
Acetoacetic acid	W	W
Formic acid	W	W
Acetic acid	W	+
Propionic acid	−	−
Inosine	−	W
myo-Inositol	+	+
D-Sorbitol	−	−
D-Mannitol	+	+
D-Arabitol	−	W
Glycerol	+	+
D-Glucose-6-PO_4_	+	+
D-Fructose-6-PO_4_	+	+
D-Aspartic acid	+	+
D-Serine	−	−
L-Serine	+	+
L-Alanine	−	W
L-Arginine	−	−
L-Aspartic acid	+	+
L-Glutamic acid	W	W
L-Histidine	−	−
L-Pyroglutamic acid	−	−
Gelatin	−	−
Glycyl-L-proline	−	−
Fatty acid content (%)		
C_12:0_	5.71	6.69
C_13:0_	0.52	0.84
C_14:0_	4.19	2.13
C_16:0_	28.75	25.55
C_17:0_	2.10	2.59
C_17:0_ cyclo	0	0.77
C_18:0_	0.56	0.47
C_15:1_ ω8*c*	0	0.19
C_16:1_ ω7*c/*C_16:1_ ω6*c*	28.96	29.44
C_17:1_ ω8*c*	0.51	0.97
C_18:1_ ω7*c*/C_18:1_ ω6*c*	18.06	19.47
C_14:0_ 3-OH/iso-C_16:1_ I/C_12:0_ aldehyde	10.39	9.29
C_15:0_ 3-OH	0.26	0.34

*+, −, and W, respectively, indicate positive, negative, and weak reaction.*

### Pan-Genome Analysis of LJ1 and LJ2

Since *P. aroidearum* LJ2 and “*P. colocasium* sp. nov.” LJ1 are close relatives but differ in their virulence against multiple hosts, we carried out pan-genome analysis to identify genes unique to either species (i.e., lack orthologs in the other species). As a result, 570 genes were found to be unique to LJ1, and 612 genes were unique to LJ2 (Supplementary Table 3). Among these, 107 LJ1 genes and 165 LJ2 genes were predicted to encode virulence factors, accounting for 18.8 and 27.0% of all unique genes in the two strains, respectively (Supplementary Table 3). Furthermore, we examined the presence of genes encoding major types of secretion systems and CWDEs in *Pectobacterium*, and the results were as follows.

#### Type 1 Secretion System

**FIGURE 4:**
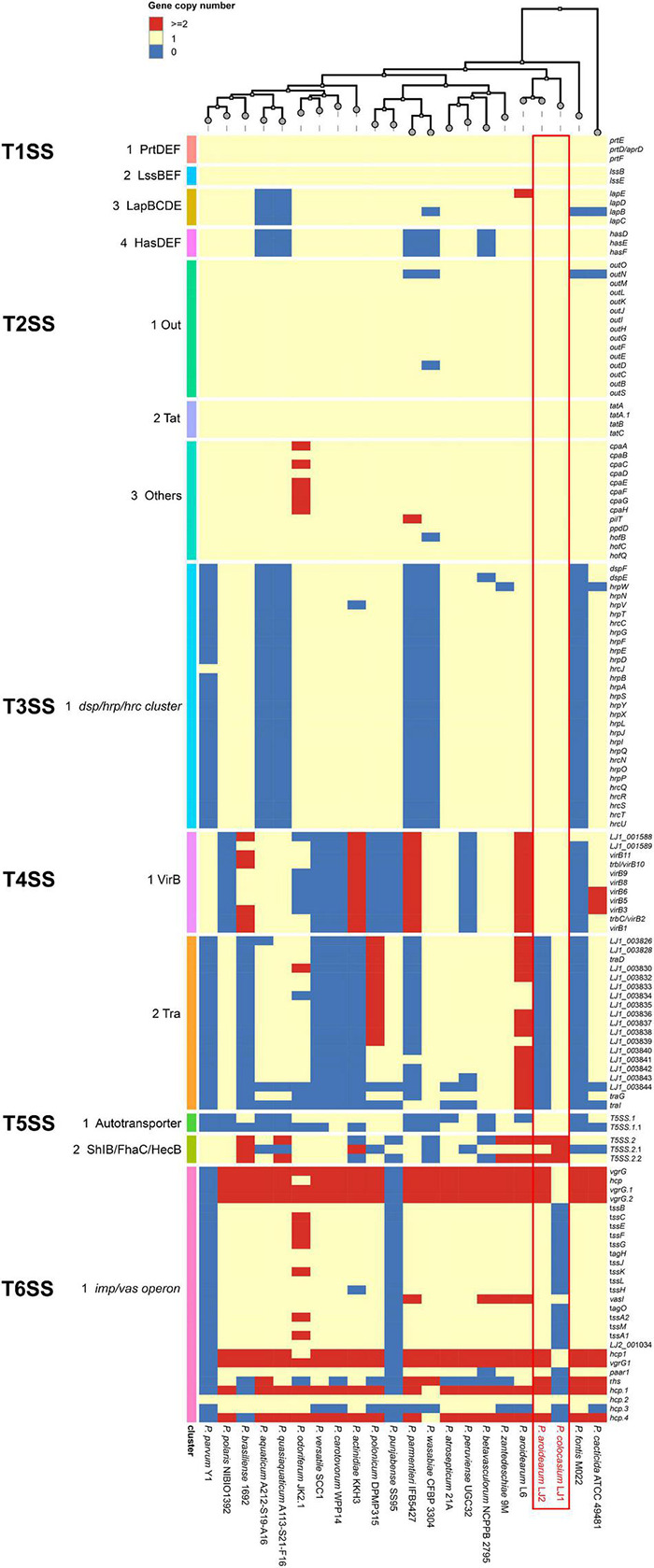
Copy numbers of genes encoding T1SS–T6SS in the genomes of *Pectobacterium colocasium* sp. nov. LJ1, *Pectobacterium aroidearum* LJ2, and 20 representative *Pectobacterium* strains. In the heatmap, each row represents a gene and each column represents a strain. The colors of blue, yellow, and red correspond to gene copy numbers of 0, 1, and 2 or more, respectively.

LJ1 and LJ2 both possess an ABC-type PrtDEF T1SS, a LssBEF T1SS, a LapBCDE T1SS, and a HasDEF T1SS (Supplementary Table 4). While the PrtDEF and LssBEF T1SSs are present in all *Pectobacterium* species, the LapBCDE and HasDEF T1SSs are missing in two and five species, respectively (Figure 4).

#### Type 2 Secretion System

LJ1 and LJ2 are both equipped with the T2SS Out secretion system and the twin-arginine translocation (Tat) protein system (Supplementary Table 4). Almost all T2SS encoding genes are universally distributed in *Pectobacterium* (Figure 4).

#### Type 3 Secretion System

**FIGURE 5:**
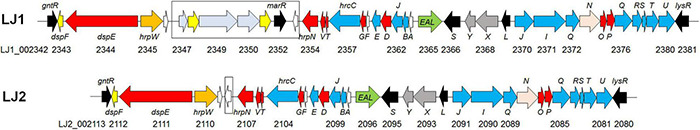
Gene arrangements of T3SS in the genomes of *Pectobacterium colocasium* sp. nov. LJ1 and *Pectobacterium aroidearum* LJ2. Genes are plotted as arrows in the order of their genomic positions in the T3SS gene cluster. The direction of each arrow indicates the gene coding strand, and the length of each arrow is in proportion to the size of the gene. Arrows in the colors of yellow, red, blue, black, and pink correspond to genes encoding molecular chaperones, effectors, structural proteins, regulators, and ATPase, respectively. The black box highlights the fimbrial gene cluster insertion in the T3SS of LJ1.

In a survey, 15 of the 21 *Pectobacerium* species share a T3SS secretion system conserved in many phytopathogenic bacteria (Figure 4 and Supplementary Table 4). The T3SS gene cluster has a conserved organization in LJ1 and LJ2, consisting of 29 genes. At the same time, LJ1 has six additional genes in between *hrpW* and *hrpN*, encoding three fimbrial proteins (*LJ1_002347*, *LJ1_002349*, and *LJ1_002350*), two molecular chaperones (*LJ1_002348* and *LJ1_002351*), and a MarR family transcriptional regulator (*LJ1_002352*) (Figure 5 and Supplementary Table 4). Similar gene clusters with conserved sequences (similarity > 70% at protein sequence level) and gene organization were only found in several strains of *Klebsiella pneumoniae*, *Providencia alcalifaciens*, *Pr. burhodogranariea*, and *Ps. otitidis* (Supplementary Table 5). Besides, these inserted genes are homologous to fimbriae-encoding genes in many bacteria. Specifically, *LJ1_002347* encodes a type 1 fimbrial protein sharing 31.43% identity/96% coverage with the minor fimbrial subunit HifD in *H. influenza* (P45992.1); *LJ1_002348* and *LJ1_002351* encode molecular chaperones, respectively, sharing 29.29% identity/95% coverage, and 35.00% identity/60% coverage with HifB (P45991.1); *LJ1_002349* encodes a fimbrial biogenesis outer membrane usher protein sharing 29.13% identity/99% coverage with HifC (P45997.1). Interestingly, a GntR and a LysR family transcriptional regulators are, respectively, located before and after the T3SS gene cluster (Figure 5).

#### Type 4 Secretion System

Both LJ1 and LJ2 harbor the same VirB family T4SS encoded by 11 *virB* genes, while LJ1 possesses a Tra type of T4SS containing 19 genes (Supplementary Table 4). The numbers of both T4SSs are highly variable among *Pectobacterium* species (Figure 4).

#### Type 5 Secretion System

Two autotransporter domain-containing proteins and three ShlB/FhaC/HecB family hemolysin secretion/activation proteins were predicted in both LJ1 and LJ2 (Supplementary Table 4). The copy numbers of these proteins are highly variable in *Pectobacterium* spp. (Figure 4).

#### Type 6 Secretion System

The genome of LJ2 harbors an integral set of T6SS encoded by genes ranging from *LJ2_001019* to *LJ2_001048*, and other 3 *vgrG* and 6 *hcp* genes, whereas LJ1 lacks the entire T6SS except for six genes, including *vasI* (*LJ1_003849*), *paar1* (*LJ1_003852*), *vgrG1* (*LJ1_003854*), *hcp1* (*LJ1_003855*), *LJ1_002759*, and *hcp* (*LJ1_003561*) genes (Supplementary Table 4 and Figure 4).

#### Cell Wall Degrading Enzymes

**FIGURE 6:**
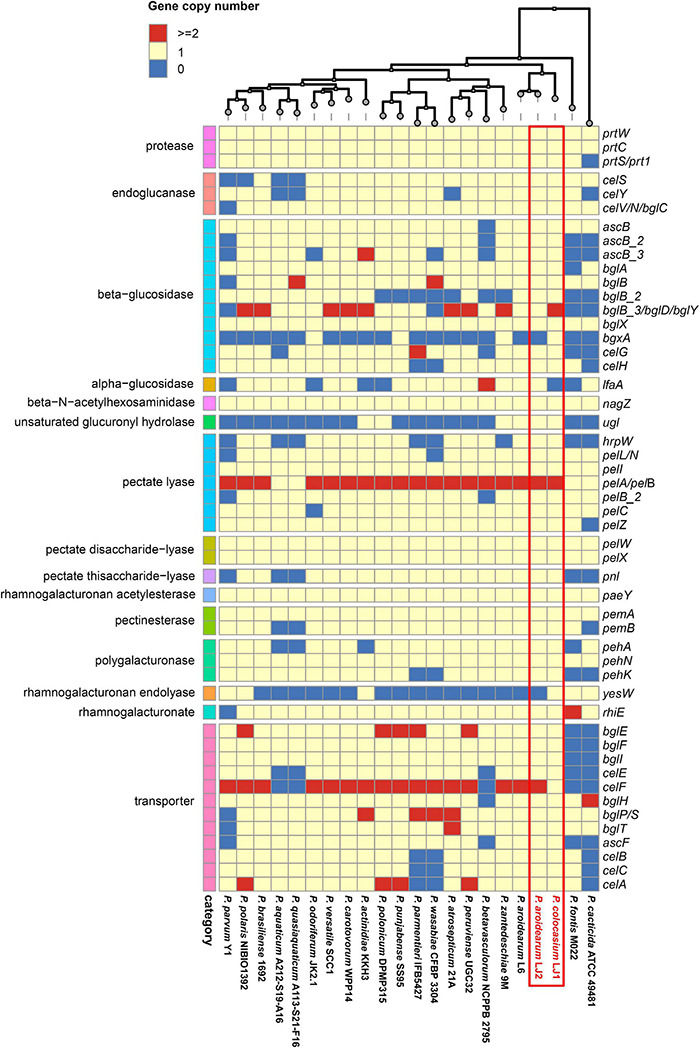
Copy numbers of CWDE-encoding genes in the genomes of *Pectobacterium colocasium* sp. nov. LJ1, *Pectobacterium aroidearum* LJ2, and 20 representative *Pectobacterium* strains. In the heatmap, each row represents a gene and each column represents a strain. The colors of blue, yellow, and red correspond to gene copy numbers of 0, 1, and 2 or more, respectively.

We looked for homologs of these known CWDEs in LJ1 and LJ2, and found that they both harbor only one copy of the serralysin protease named PrtW, adjacent to the T1SS, while four copies (PrtX, PrtC, PrtB, and PrtG) were found in *D. dadantii* 3937, *D. oryzae* EC1, and *D. zeae* MS2 (Zhou et al., 2015; Hu et al., 2022). The protein sequences of PrtW share 93.29% identity to each other in strains LJ1 and LJ2. Besides, PrtC and PrtS (Prt1), sharing high amino acid similarity to each other, were also predicted in both genomes (Supplementary Table 6 and Figure 6). Interestingly, although LJ1 and LJ2 share a highly conserved repertoire of proteases, the protease activity of LJ2 is significantly stronger than that of LJ1 (Supplementary Figure 4).

Eighteen genes encoding cellulose degradation enzymes were identified in LJ1 and/or LJ2, including 3 endoglucanase encoding genes (*celS*, *celY*, and *celV*), 12 beta-glucosidase encoding genes (*acsB*, *acsB_2*, *acsB_3*, *bglA*, *bglB*, *bglB_2*, *bglB_3*, *bglD*, *bglX*, *bgxA*, *celG*, and *celH*), an alpha-glucosidase encoding gene (*lfaA*), a beta-N-acetylhexosaminidase encoding gene (*nagZ*), and an unsaturated glucuronyl hydrolase encoding gene (*ugl*) (Supplementary Table 6 and Figure 6). Among these genes, *lfaA* is absent in LJ1, while *bglB_3* and *bgxA* are absent in LJ2 (Supplementary Table 6 and Figure 6). The cellulose-degrading activities are similar in LJ1 and LJ2 (Supplementary Figure 4).

Both LJ1 and LJ2 contain a total of 19 genes encoding pectin degradation enzymes, including eight pectate lyase encoding genes (*hrpW*, *pelL*/*pelN*, *pelI*, *pelA*, *pelB*, *pelB_2*, *pelC*, and *pelZ*), two pectate disaccharide-lyase encoding genes (*pelW* and *pelX*), a pectate thisaccharide-lyase encoding gene (*pnl*), a rhamnogalacturonan acetylesterase encoding gene (*paeY*), three pectinesterases encoding genes (*pemA* and *pemB*), three polygalacturonase encoding genes (*pehA*, *pehN*, and *pehK*), a rhamnogalacturonan endolyase encoding gene (*yesW*), and a rhamnogalacturonate lyase encoding gene (*rhiE*) (Supplementary Table 6 and Figure 6). LJ2 produced slightly reduced pectin degrading activity compared with LJ1 (Supplementary Figure 4).

## Discussion

Soft rot Pectobacteriaceae (SRP) are among the most important bacterial plant pathogens, causing huge economic losses in the production of many crops, vegetables, and ornamentals (Mansfield et al., 2012). SRP are highly diverse and complex in that many of the pathogens have overlapping host ranges and geographical distributions. Moreover, there are frequent reports of novel SRP pathogens or new hosts of known SRP pathogens. The dominant SRP pathogens for a given disease can also vary over time, possibly due to the changes in host cultivars and environmental conditions and the evolution of pathogens themselves. For instance, *D. oryzae* (formerly belonged to *D. zeae*) has been the primary pathogen causing soft rot disease on crops in south China since the 1980s (Hong et al., 1983; Yang, 2000; Lin and Feng, 2006). In recent years, however, *D. zeae* and *D. fangzhongdai* are more frequently isolated from crops, fruit trees, vegetables, ornamentals, and even herbaceous plants (Lin et al., 2010; Zhang et al., 2014; Hu et al., 2018; Shen et al., 2019; Chen et al., 2020; Boluk et al., 2021; Huang et al., 2021; Wang et al., 2021). Therefore, the success of control efforts against SRP diseases depends on a good understanding of the pathogens and their pathogenicity-related characteristics.

In this study, we investigated the pathogens of soft rot disease of Pinang taro in Ruyuan County, Shaoguan City, China. Previously only *D. zeae* and *D. fangzhongdai* have been shown to be causative pathogens of bacterial taro soft rot (Boluk et al., 2021; Huang et al., 2021). A few reports stated that *P. carotovorum* is also a pathogen of taro (e.g., Wu and Dai, 2015; Dong et al., 2021), yet no experimental evidence for the isolation and identification was provided. Here, we identified two *Pectobacterium* strains, namely LJ1 and LJ2, as causal agents of the disease through pathogen isolation, pathogenicity assay, and genomic sequencing and analysis. While LJ2 belongs to *P. aroidearum*, LJ1 shares relatively low levels of genomic similarity (ANI ≤ 93.68%; dDDH ≤ 52.00%) with known *Pectobacterium* species, and has different biochemical and chemotaxonomic characteristics from its closest relative *P. aroidearum*. Therefore, LJ1 represents a novel species which we propose to name “*Pectobacterium colocasium* sp. nov.” Overall, our results add *Pectobacterium* to the list of SRP pathogens of taro soft rot, discover “*P. colocasium* sp. nov.” as a new member of the genus, and expand the host range of *P. aroidearum* to include taro.

Although SRP pathogens are frequently found to co-exist in the same fields (Terta et al., 2010; Oulghazi et al., 2017; van der Wolf et al., 2017; Naas et al., 2018; Ozturk et al., 2018; Portier et al., 2019), so far there are only a few reported cases of co-infection (Ge et al., 2021; Hu et al., 2021a; Motyka-Pomagruk et al., 2021). In this study, LJ1 and LJ2 were isolated from the same soft rot taro corm; the two pathogens can infect taro both individually and together, although LJ2 caused more severe disease symptoms than LJ1 at 48-hpi (Figure 2). Moreover, the co-infection of LJ2 with LJ1 did not alter the disease progression or the accumulation of either strain *in planta* (Figure 2). Together, our results suggest that: (1) both LJ1 and LJ2 are pathogens contributing to the soft rot of taro in Ruyuan County; (2) LJ2 is more virulent than LJ1 at later stages of infection; and (3) there is no obvious interaction between LJ1 and LJ2. In future studies, it would be of interest to carry out broader surveys of the prevalence of the two pathogens, and to monitor the long-term interaction between LJ1 and LJ2 (and potentially other taro soft rot pathogens such as *D. fangzhongdai*) under field conditions.

It is worth noting that, although LJ1 and LJ2 were both isolated from taro, host ranges of LJ1 and LJ2 might also encompass eudicots. Our virulence assays showed that LJ1 and LJ2 can also infect potato, Chinese cabbage, and celtuce; LJ2 was even more virulent than *P. carotovorum* BC9 on cabbage and celtuce (Supplementary Figure 1). On the other hand, interestingly, both strains caused only slight disease symptoms on *Alocasia macrorrhizos* (giant taro), which is a close relative of taro (Supplementary Figure 1). The host range of SRP pathogen is affected by multiple factors such as the pathogen virulence, pathogen-host interaction, and environmental conditions. Some SRP pathogens may exhibit broad host ranges in laboratory studies, but have only been isolated from limited hosts in nature (Hu et al., 2018). Recent studies have started to examine the host range and host specialization of *Pectobacterium* pathogens (Khadka et al., 2020), but more efforts are needed to systematically investigate this issue.

Pan-genome analysis of LJ1, LJ2, and other *Pectobacterium* allowed us to better understand the molecular basis of their pathogenicity. In comparing the two taro soft rot pathogens, we found 570 genes unique to LJ1 and 612 genes unique to LJ2, many of which were predicted to encode virulence factors. Notably, LJ2 has 58 more unique predicted virulence factors than LJ1, including an extra set of T6SS, several type VI secreted effectors, and 20 transcription regulators (vs. only three among LJ1 unique virulence factors) (Supplementary Table 2). These strain-specific virulence factors might contribute to the greater virulence of LJ2. In addition, we also found other genomic features of LJ1 and LJ2 that might be of significance in shaping their pathogenicity. For instance, our analysis of secretion systems found an insertion of a fimbrial gene cluster (*LJ1_002347*-*LJ1_002353*) in the T3SS gene cluster that is uniquely present in the genome of LJ1 (Figure 5). Five of the inserted genes are homologous to genes encoding type 1 and type 3 fimbrae in many animal pathogens, such as *Haemophilus influenza* (van Ham et al., 1989, 1994), *Escherichia coli* (Klemm and Christiansen, 1990), and *Stenotrophomonas maltophilia* (de Oliveira-Garcia et al., 2003). The fimbriae encoded by the *hif* gene cluster in *H. influenza* and the *fim* gene cluster in *E. coli* and *S. maltophilia* have been characterized to mediate attachment on the host cells, often contributing to virulence and disease (van Ham et al., 1989, 1994; Klemm and Christiansen, 1990; de Oliveira-Garcia et al., 2003). Highly similar gene clusters are only found in certain strains of *K. pneumoniae*, *Pr. alcalifaciens*, and *Ps. otitidis*, suggestive of horizontal gene transfer, which has been considered an important mechanism for Pectobacteria to acquire virulence determinants (Davidsson et al., 2013). However, it remains to be determined if the role of this fimbrial gene cluster in the virulence of LJ1 is positive or negative, as its insertion might disrupt the T3SS.

In addition, LJ1 contains an extra set of *tra*-T4SS (*LJ1*_003823-*LJ1*_003848) compared to LJ2 (Supplementary Table 4), which is present in many other *Pectobacterium* strains, as well as *D. dadantii* M2-3 and S3-1, *D. zeae* MS2, and *D. chrysanthemi* Ech1591. On the other hand, analysis of the T6SS revealed that LJ1 lost almost the whole *imp/vas* operon encoding the secretion machinery of T6SS with only four genes left, including *vasI* (*LJ1_003849*), *paar1* (*LJ1_003852*), *vgrG1* (*LJ1_003854*), and *hcp1* (*LJ1_003855*). VgrG and Hcp are secreted by the T6SS secretion machinery along with Rhs effector proteins and form a membrane puncturing device. SRP possess variable numbers of *hcp-vgrG-rhs* clusters and T6SS. For example, *P. wasabiae* and *P. parmentieri* harbor two copies of T6SS genes with different gene organizational patterns.

As “brute-force” pathogens, SRP utilize plant CWDEs as weapons to intrude plant cell walls by degrading plant tissues and disintegrating plant cell structures. A battery of CWDEs (e.g., proteases, cellulases, and pectate lyases) and their global regulators (e.g., KdgR, PecS, H-NS, Fis, SlyA, PecT, and VfmE) have been well-characterized in *Dickeya* spp. (Nasser et al., 1991, 2013; Reverchon et al., 1994; Ouafa et al., 2012; Hérault et al., 2014; Zhou et al., 2016; Lv et al., 2018, 2019). *Pectobacterium* also produces a variety of plant CWDEs, especially pectate lyases, as the major virulence factors causing plant tissue decay and maceration (Toth et al., 2003). Genomic comparison of LJ1 and LJ2 revealed a highly conserved repertoire of CWDE-encoding genes except for the loss of the alpha-glucosidase encoding gene *lfaA* in LJ1, and the loss of the alpha-glucosidase encoding gene *bgxA* and rhamnogalacturonan endolyase encoding gene *yesW* in LJ2 (Supplementary Table 6). Interestingly, we found that LJ2 produced fewer pectinases but more proteases than LJ1, despite the fact that they have the same repertoire of proteases, pectinases, and related transporters. One possible explanation is that the CWDE-encoding genes are differentially regulated in the two pathogens. We recently studied *D. zeae* MS2 and JZL7 and found that, while the two strains have almost the same set of CWDE-encoding genes, JZL7 produces remarkably less amount of CWDEs due to lower gene expression levels (Hu et al., 2022).

Overall, the pathogenicity of SRP is an integrated outcome of many factors such as regulatory mechanisms (e.g., quorum sensing) (Charkowski et al., 2012), secretion systems (Rantakari et al., 2001), virulence factors, motility (Matsumoto et al., 2003), biofilms (Lee et al., 2013), etc. The two newly identified *Pectobacterium* pathogens of taro and the genomic data generated in this study represent valuable resources for further investigation of the pathogenic mechanisms of SRP, and shed insights into the development of disease control strategies. The production of PCWDEs is controlled by different QS systems between *Pectobacterium* and *Dickeya* (Liu et al., 2022). The AHL QS system is crucial in the production of PCWDEs and other virulence factors in *Pectobacterium* (Barnard and Salmond, 2007; Liu et al., 2008), but has little impact on the total pectate lyase activity in *Dickeya* (Nasser et al., 1998; Liu et al., 2022). Instead, the *Dickeya*-specific QS system VFM plays a key role in regulating the production of PCWDEs (Nasser et al., 2013; Lv et al., 2019; Liu et al., 2022). Therefore, in the control of taro soft rot, strategies (e.g., quorum-quenching biocontrol agents) targeting the VFM QS system will likely give rise to better efficacy for *Dickeya* spp. pathogens, while the AHL QS signal would be a more suitable target if the disease is caused by *Pectobacteium* spp.

## Data Availability Statement

The datasets presented in this study can be found in online repositories. The names of the repository/repositories and accession number(s) can be found in the article/Supplementary Material.

## Author Contributions

JZ conceived and designed the experiments and wrote the manuscript. MH, YXue, CT, and YXu collected the diseased taro plants and performed the disease survey. MH, XR, MT, YXue, and SC isolated and identified the pathogens. MH and AH performed the antagonistic assay and the pathogenicity tests. CL and XZ analyzed the genome data. MH, LZ, and XZ revised the manuscript. All authors contributed to the article and approved the submitted version.

## Conflict of Interest

CT was employed by Guangdong Tianhe Agricultural Means of Production Co., Ltd. YXu was employed by Guangdong Tianhe Agricultural Means of Production Co., Ltd. and Qingyuan Agricultural Science and Technology Service Co., Ltd. The remaining authors declare that the research was conducted in the absence of any commercial or financial relationships that could be construed as a potential conflict of interest.

## Publisher’s Note

All claims expressed in this article are solely those of the authors and do not necessarily represent those of their affiliated organizations, or those of the publisher, the editors and the reviewers. Any product that may be evaluated in this article, or claim that may be made by its manufacturer, is not guaranteed or endorsed by the publisher.

## Footnotes

1
http://gdnykx.cnjournals.org/html/2021/6/202106017.html

2
http://ggdc.dsmz.de/
