# Supplementary material for: Isolation and Genome Analysis of Pectobacterium colocasium sp. nov. and Pectobacterium aroidearum, Two New Pathogens of Taro
Source: Front Plant Sci. 2022 Apr 26;13:852750. doi: 10.3389/fpls.2022.852750 (PMC9088014; doi:10.3389/fpls.2022.852750)
Supplement: Supplementary file 2 [file Data_Sheet_1.doc]

**Supplemental Material**

**Contents:**

**Supplementary Table S1**

**Supplementary Table S6**

**Supplementary Fig. S1**

**Supplementary Fig. S2**

**Supplementary Fig. S3**

**Supplementary Fig. S4**

**Table S1.** Subject organisms of the isolates from diseased taro corms based on 16S rDNA sequences

| **Query strain** | **Subject strain** | **Coverage** | **Identity** |
| --- | --- | --- | --- |
| LJ1, LJ3 | *Pectobacterium carotovorum* subsp. *carotovorum* PCCS1 | 100% | 99.20% |
| LJ2, LJ4, LJ5, LJ6, LJ7, LJ8, LJ9, LJ10 | *Pectobacterium aroidearum* L6 | 100% | 99.73% |
| L11, L12 | *Klebsiella pneumoniae* NPK3_1_46 | 99% | 99.54% |
| L13 | *Klebsiella pneumoniae* EB20-THQ | 99% | 99.62% |
| L14 | *Weissella cibaria* 2781 | 99% | 99.44% |
| L15 | *Bacillus aryabhattai* SCSGAB0134 | 99% | 99.53% |
| L16 | *Bacillus* sp. (in: Bacteria) Z64 | 99% | 99.45% |
| L17 | *Bacillus megaterium* ANA29 | 99% | 99.34% |
| L18 | *Bacillus cereus* K1M16 | 99% | 99.61% |

**Table S6. Cell wall degrading enzymes predicted in the genomes of strains LJ1 and LJ2**

| **Gene** | **LJ1** | **LJ2** | **Identity (%)** | **Product** |
| --- | --- | --- | --- | --- |
| *prtC* | LJ1_000667 | LJ2_003659 | 96.91 | U32 family peptidase |
| *prtW* | LJ1_001748 | LJ2_002708 | 93.29 | M10 family metallopeptidase |
| *prtS/1* | LJ1_003138 | LJ2_001246 | 97.69 | Protease PrtS, peptidase M4 family protein |
| *bglA* | LJ1_000012 | LJ2_000012 | 98.54 | 6-phospho-beta-glucosidase |
| *celG* | LJ1_000036 | LJ2_000033 | 97.42 | 6-phospho-beta-glucosidase |
| *celY* | LJ1_000071 | LJ2_000086 | 94.88 | Cellulase |
| *ascB* | LJ1_000612 | LJ2_003716 | 98.7 | 6-phospho-beta-glucosidase |
| *bglB_3* | LJ1_000847 | / | / | Glycoside hydrolase family 1 protein |
| *bglD/Y* | LJ1_001436 | LJ2_002975 | 97.47 | Glycoside hydrolase family 1 protein, beta-glucosidase |
| *ascB_3* | LJ1_001461 | LJ2_002952 | 97.33 | Family 1 glycosylhydrolase |
| *bglB_2* | LJ1_001554 | LJ2_002832 | 97.7 | 6-phospho-beta-glucosidase |
| *celS* | LJ1_001696 | LJ2_002758 | 92.8 | Endoglucanase CelS |
| *bglX* | LJ1_001743 | LJ2__002713 | 98.83 | Beta-glucosidase BglX |
| *lfaA* | / | LJ2_001952 | / | Glycoside hydrolase family 31 protein |
| *celV/N/bglC* | LJ1_002475 | LJ2_001968 | 96.44 | Endoglucanase N, cellulase family glycosylhydrolase CelV |
| *bglB* | LJ1_002574 | LJ2_001859 | 98.49 | 6-phospho-beta-glucosidase |
| *nagZ* | LJ1_002621 | LJ2_001808 | 95.03 | Beta-N-acetylhexosaminidase |
| *ascB_2* | LJ1_002284 | LJ2_002148 | 98.74 | 6-phospho-beta-glucosidase |
| *bgxA* | LJ1_003368 | / |  | Periplasmic beta-glucosidase |
| *celH* | LJ1_003525 | LJ2_000813 | 97.49 | Glycoside hydrolase family 1 protein |
| *ugl* | LJ1_004055 | LJ2_000308 | 91.82 | Glycoside hydrolase family 88 protein |
| *rhiE* | LJ1_000781 | LJ2_003572 | 92.01 | Rhamnogalacturonate lyase |
| *pelI* | LJ1_001039 | LJ2_003316 | 96.01 | Pectate lyase |
| *pehA* | LJ1_001040 | LJ2_003315 | 96.02 | Endopolygalacturonase |
| *pehN* | LJ1_001140 | LJ2_003213 | 98.04 | Polygalacturonase PehN |
| *pnl* | LJ1_001479 | LJ2_002929 | 95.86 | Pectate trisaccharide-lyase |
| *pelL/N* | LJ1_001934 | LJ2_002501 | 97.25 | Pectate lyase PelL |
| *pelW* | LJ1_002067 | LJ2_002366 | 98.34 | Pectate disaccharide-lyase |
| *pelB* | LJ1_002317 | LJ2_002120 | 98.24 | Periplasmic pectate lyase |
| *hrpW* | LJ1_002345 | LJ2_002110 | 97.47 | Pectate_lyase, type III effector protein |
| *paeY* | LJ1_003178 | LJ2_001206 | 94.75 | Rhamnogalacturonan acetylesterase |
| *pemA* | LJ1_003179 | LJ2_001205 | 94.02 | Pectinesterase A |
| *pehK* | LJ1_003438 | LJ2_000906 | 92.8 | Polygalacturonase PehK |
| *yesW* | LJ1_003611 | / | / | Rhamnogalacturonan endolyase YesW |
| *pelA* | LJ1_003967 | LJ2_000407 | 98.13 | Pectate lyase PelA, polysaccharide lyase |
| *pelB* | LJ1_003968 | LJ2_000406 | 97.06 | Pectate lyase PelB, polysaccharide lyase |
| *pelC* | LJ1_003969 | LJ2_000405 | 98.13 | Pectate lyase PelC,polysaccharide lyase |
| *pelZ* | LJ1_003970 | LJ2_000404 | 94.43 | Pectate lyase PelZ |
| *pemB* | LJ1_004262 | LJ2_004208 | 86.75 | Pectin methylesterase PemB |
| *pelX* | LJ1_004383 | LJ2_004326 | 97.18 | Pectate disaccharide-lyase |

**
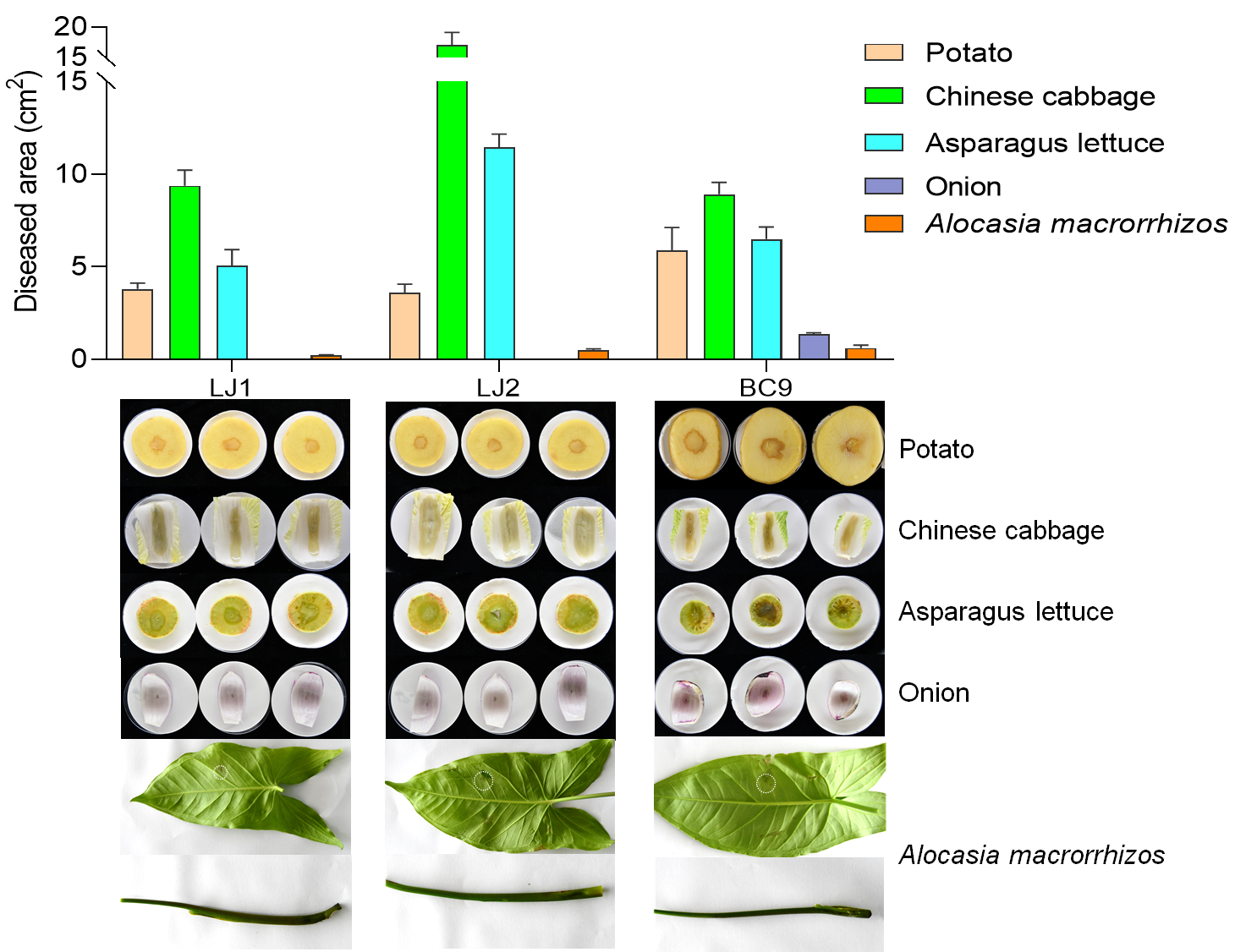
**

**Fig. S1.** Measurement of host spectrum of LJ1 and LJ2. Equal volume of LJ1, LJ2, and positive control of *Pectobacterium carotovorum* subsp. *carotovorum* BC9 was inoculated onto the plant materials with methods provided in the text.

**
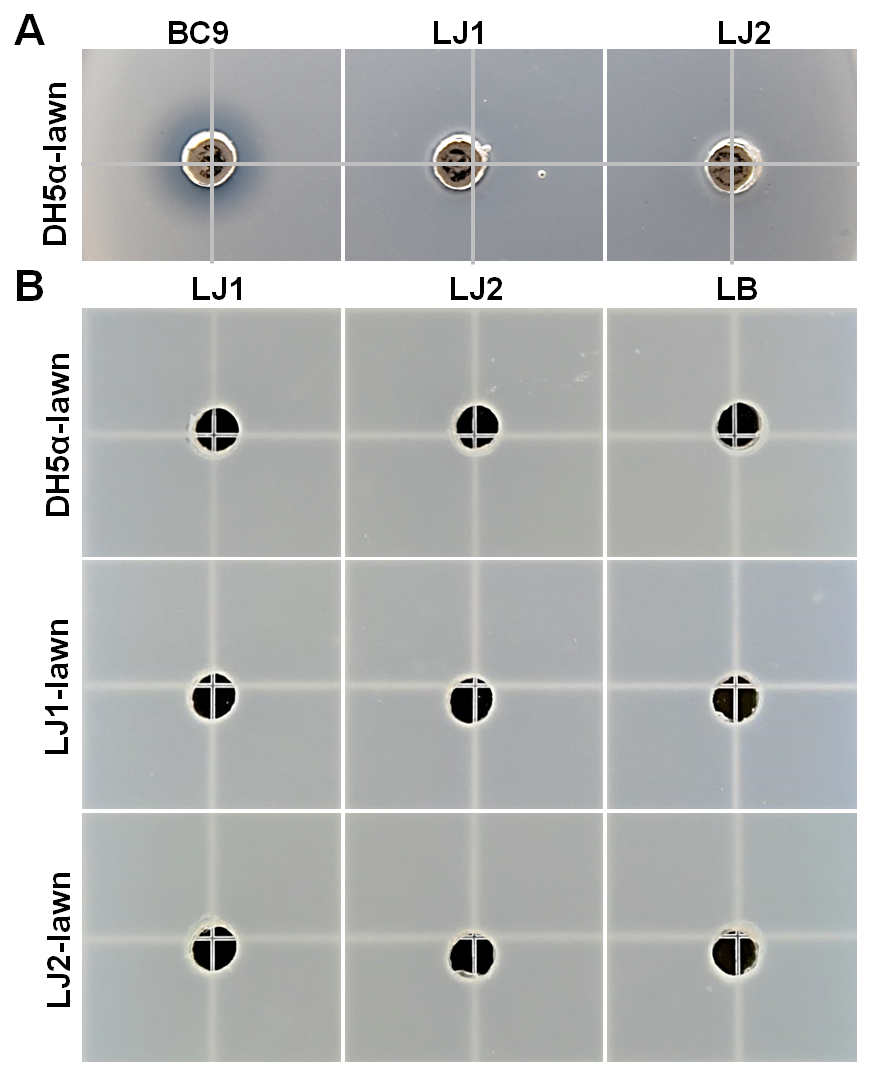
**

**Fig. S2.** Measurement of antagonism between isolates LJ1 and LJ2. **A.** Antagonism of different *Pectobacterium* spp. against *E. coli* DH5α. **B.** Antagonism between LJ1 and LJ2. Spot-on-lawn assay was performed to determine whether there is any antagonistic interaction between isolates LJ1 and LJ2 isolated from the same diseased taro sample. In brief, 1 mL of LJ1, LJ2 or *E. coli* DH5α bacterial cultures (OD_600_=1.2) was added into 20 mL of 1% melted agarose (cool to 50~60 ℃), mixed thoroughly and then laid onto a LB agar plate (10 cm × 10 cm). After dryness, wells of 5 mm in diameter were punched and applied with 20 μL of the bacterial cultures. The plates were incubated at 28 ℃ for 24 h. LB medium was used as the negative control. The experiment was repeated three times in triplicate.

**
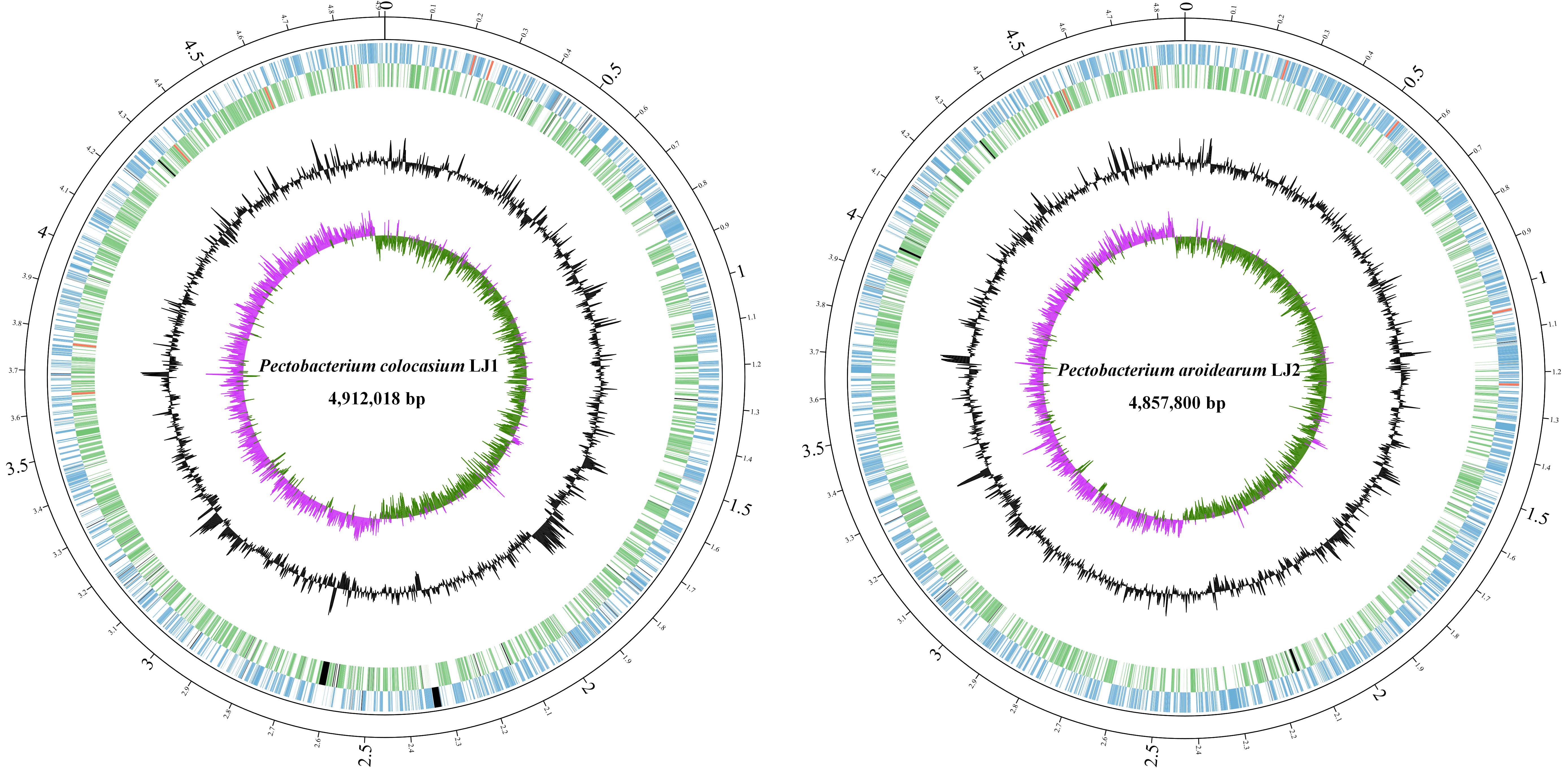
**

**Fig. S3. Circular genome maps of *Pectobacterium colocasium* sp. nov. LJ1 and *Pectobacterium aroidearum* LJ2.** The circles from outside to inside represent features on the positive strand: CDS (blue), rRNA (red), pseudogene (black); features on the negative strand: CDS (green), rRNA (red), pseudogene (black); GC content; and GC-skew value (green/pink).


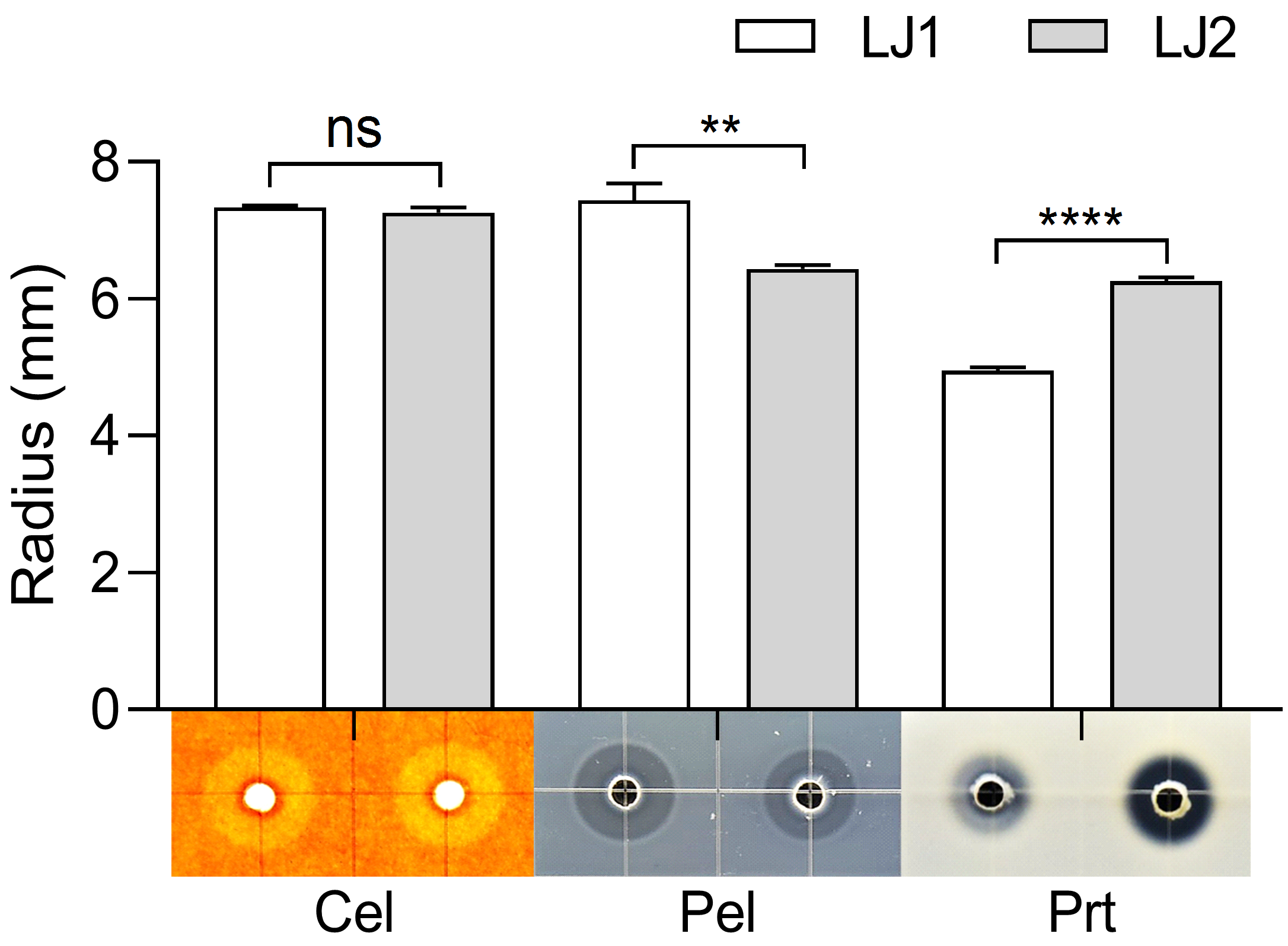


**Fig. S4. Extracellular cell wall degrading enzymes (CWDEs) produced by strains LJ1 and LJ2.** The activities of CWDEs were measured using the assay medium recipe described previously (Zhou et al., 2016). GraphPad Prism 8.4.1 was used to performed unpaired two-tailed *t*-test, and the data of strain LJ2 were compared with those of strain LJ1. The data present the means of three replicates and error bars represent the standard deviation. "ns" indicates not significant, ** indicates P<0.01, and **** indicates P<0.0001.

Reference:

Zhou, J., Zhang, H., Lv, M., Chen, Y., Liao, L., Cheng, Y., et al. (2016). SlyA regulates phytotoxin production and virulence in *Dickeya zeae* EC1. Mol. Plant Pathol. 17:1398-1408.
